# Blind placement of postpyloric feeding tubes at the bedside in intensive care

**DOI:** 10.1186/s13054-021-03587-5

**Published:** 2021-05-11

**Authors:** Qianwen Wang, Yongbo Xuan, Cuiping Liu, Mei Lu, Zhanguo Liu, Ping Chang

**Affiliations:** Department of Critical Care Medicine, Zhujiang Hospital, Southern Medical University, 253 Gongye Middle Avenue, Haizhu District, Guangzhou, 510280 Guangdong China

Postpyloric feeding is recommended for those who cannot tolerate gastric enteral nutrition or who are at high risk of aspiration [1–3]. This approach can reduce respiratory and gastrointestinal complications and achieve nutritional goals earlier and more effectively. A large cohort study that investigated the nutritional support habits in the intensive care unit (ICU) revealed that the nasojejunal tube was only applied in 5.5% of the patients [4]. The lack of effective transpyloric placement methods may be a critical reason for the low application of nasojejunal tube. Various blind techniques for postpyloric feeding tube placement have been applied to clinical practice. Unfortunately, no unified opinion exists for these techniques. Several studies state that the success rate of blind placement ranged from 35% to 100%. Consequently, blind placements using the Corpak postpyloric feeding tube may be another alternative approach. Andrew et al. reported that the best success rate was 90% [5]. However, only 20 patients with gastric ileus were enrolled in their study. This retrospective study evaluated the safety and efficiency of blind bedside postpyloric placement and investigated the potential risk factors influencing the placement in critically ill patients.

The study protocol was approved by Zhujiang Hospital Ethical Committees (2020-KY-064-01). Patients who underwent blind bedside insertion of Corpak postpyloric feeding tube between December 2016 and January 2020 were included in Department of Critical Care Medicine. This operation was performed by experienced head nurses or nurse leaders. For patients without any contraindications, 10 mg of metoclopramide was administrated before the intubation. Upper abdominal radiography was requested to confirm the position of the tube tip within 24 h. The primary outcome was the success rate of placement. The success rates of post-third portion of the duodenum (D3), post-fourth portion of the duodenum (D4), proximal jejunum placement, insertion length, time for insertion, number of attempts, and the possible risk factors for tube placement failure were secondary outcomes. Safety endpoints were major tube-associated and metoclopramide-related adverse events.

The postpyloric placement was achieved in 83.7% (236/282) of patients, with 69.9% (197/282) of the patients completed in the first attempt. The success rates of post-D3, post-D4, and proximal jejunum placement were 68.8%, 59.2%, and 25.9%, respectively. The mean length of insertion was 101.4 cm and the median time to insertion was 30 min, with 1.0 median number of attempts. These data are summarized in Table 1. Logistic regression analysis identified the use of vasopressor, patients with neurological diseases, Acute Physiology and Chronic Health Evaluation (APACHE) II score ≥ 20, Sequential Organ Failure Assessment (SOFA) score ≥ 12, Acute Gastrointestinal (AGI) grade ≥ II, and with mechanical ventilation or continuous renal replacement therapy (CRRT) as independent risk factors influencing the success rate of placement (Fig. 1). The presence of above factors indicated the critical condition of the patients and the impaired state of their gastrointestinal function. Therefore, these patients always showed a lower success rate. On the contrary, patients without the above risk factors were more likely to show successful outcomes. The adverse event incidence in this study was 2.8%. Fortunately, no severe adverse events occurred. Nasal mucosa bleeding was the most frequent major tube-associated adverse events with an incidence rate of 1.8%. However, the metoclopramide-related adverse event was not observed.

**Table 1:** The primary outcomes and secondary efficacy outcomes

Outcomes	Value in total study sample (*n* = 282)
*Primary outcomes*	
Post-pyloric placement^a^	236 (83.7%)
*Secondary outcomes*	
Placed at D3 or beyond^b^	194 (68.8%)
Placed at D4 or beyond^c^	167 (59.2%)
Placed at the proximal jejunum	73 (25.9%)
Time to insertion, min	30 (20–30)
Number of attempts	1 (1–2)
Length of insertion (cm)	101.4 ± 7.5

According to whether the variables comply with the normal distribution, quantitative variables are presented as mean ± SD or median (IQR) as appropriate and qualitative variables as numbers (percentage)

^a^Post-pyloric placement, reaching the first portion of the duodenum or beyond

^b^D3 is the third portion of the duodenum

^c^D4 is the forth portion of the duodenum

**Fig. 1:**
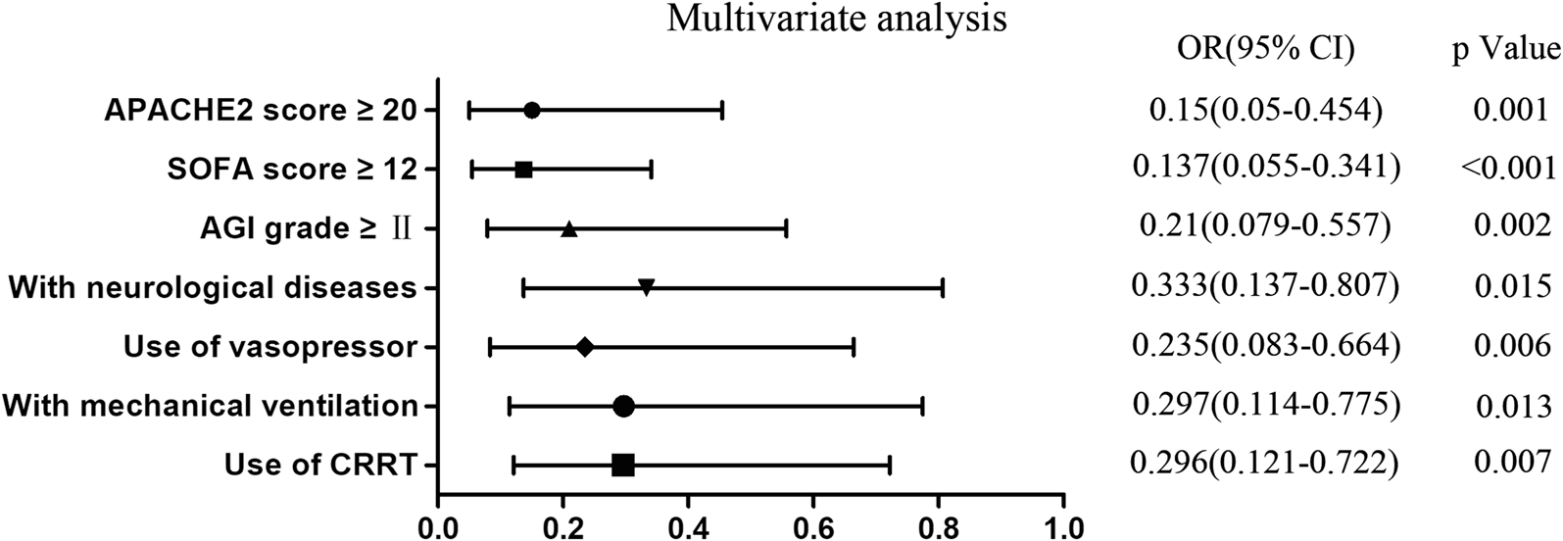
Multivariate logistic regression analysis of factors for the success of postpyloric placement. *OR* odds ratio, *CI* confidence interval; *P* < 0.05 was considered to be statistically significant

In conclusion, blind placement of Corpak postpyloric feeding tubes at the bedside was considered to be safe and effective for critically ill patients, and the results of the current study further confirmed that all the aforementioned factors were independent risk factors and the findings of this may provide evidence for tailored therapy. Thus, this technique may facilitate the establishment of postpyloric feeding in the ICU.

## Data Availability

The datasets generated and analyzed during the current study are available from the corresponding author on reasonable request.

## Abbreviations

APACHE II: Acute physiology and chronic health evaluation II
SOFA: Sequential organ failure assessment
AGI: Acute gastrointestinal injury
CRRT: Continuous renal replacement therapy
OR: Odds ratio
CI: Confidence interval
